# Retrospective Analysis of the Impact of Vaccination with an Inactivated Vaccine on Toxoplasmosis-Associated Mortality in Captive Wildlife

**DOI:** 10.3390/vaccines13090910

**Published:** 2025-08-27

**Authors:** Angelo Scuotto, Daniela Ogonczyk-Makowska, Alicia Quiévy, Mélanie Berthet, Kévin Schlax, Didier Boussarie, Alexis Maillot, Florine Popelin-Wedlarski, Thomas Charpentier, Maïalen Perot, Benoît Quintard, Marloes van Elderen, Job Benjamin Gérard Stumpel, Stamatios Alan Tahas, Anna Modlinska, Viktória Sós-Koroknai, Alexandre Azevedo, María del Carmen Carmona Muciño, Mariana Castilho Martins, Carlos Madrid, Juliana Peña Stadlin, Lina M. Henao-Montoya, Didier Betbeder

**Affiliations:** 1 Vaxinano SAS, 84 rue du Dr. Yersin, 59120 Loos, France; 2 Pairi Daiza Zoo, Domaine de Cambron, 7940 Brugelette, Belgium; 3 Parc Zoologique du Museum de Besançon, La Citadelle, 25000 Besançon, France; 4 Parc d’Isle, 02100 Saint-Quentin, France; kevin.schlax@gmail.com; 5 La Réserve Exotique (The Exotic Garden, Foster’s Center For Small Primates)—Blérancourt, 02300 Blérancourt, France; 6 L’Académie Vétérinaire de France, 75011 Paris, France; 7 Amnéville Zoo, 57360 Amneville, France; alexis@zoo-amneville.com; 8 Bioparc de Doué-La-Fontaine, 49700 Doué-la-Fontaine, France; fwedlarski@bioparc-zoo.fr; 9 La Palmyre Zoo, 17570 Les Mathes, France; veto@zoo-palmyre.com; 10 Biotropica Zoo, La Coudrette, 27100 Val de Reuil, France; 11 Zoo and Botanical Garden of Mulhouse, 68100 Mulhouse, France; 12 Apenheul Zoo, J.C. Wilslaan, 7313 HK Apeldoorn, The Netherlands; 13 Wildlands, 7811 AP Emmen, The Netherlands; 14 Copenhagen Zoo, 2000 Frederiksberg, Denmark; st@zoo.dk; 15 Curraghs Wildlife Park, Ballaugh IM7 5EA, Isle of Man; anna.modlinska@iomvet.co.uk; 16 Budapest Zoo and Botanical Garden, 1146 Budapest, Hungary; 17 Lagos Zoo, 8600-013 Lagos, Portugal; ax.c.azevedo@gmail.com; 18 CIVG—Vasco da Gama Research Center, EUVG—Vasco da Gama University School, 3020-210 Coimbra, Portugal; 19 Africam Safari Zoo, Puebla 72960, Mexico; 20 Quinzinho de Barros Zoo, Sorocaba 18020-268, SP, Brazil; marianacm.vet@gmail.com; 21 Parque de la Conservación of Médellin, Medellín 050024, Colombia; carlosmadrid@parquedelaconservacion.com; 22 Cali Zoo, FZC Fundación Zoológica de Cali, Cali 760045, Colombia; 23 Laboratorio de la Fundación Botánica y Zoológica de Barranquilla, Barranquilla 080001, Colombia

**Keywords:** *T. gondii*, toxoplasmosis, inactivated vaccine

## Abstract

**Background/Objectives**: *Toxoplasma gondii* is a major cause of zoonotic infections in both humans and animals, resulting in significant mortality in susceptible species, such as New World primates and marsupials. Toxoplasmosis is particularly concerning in zoos and wildlife reserves, where outbreaks threaten conservation efforts for endangered species. In the absence of a commercially available vaccine against toxoplasmosis for humans and captive wild animals, current prevention strategies are limited to restricting the access of cats to enclosures, controlling rodent populations, and maintaining strict food hygiene. Recent research has shown promising results with an intranasal vaccine (VXN-Toxo) composed of maltodextrin nanoparticles conjugated with a purified, inactivated *T. gondii* parasite. This experimental vaccine does not pose a risk of causing disease and offers advantages such as better stability compared with live pathogen-based vaccines. **Methods**: This study presents a large-scale evaluation of the effect of VXN-Toxo administered to captive wildlife across 20 zoos in Europe and the Americas between 2017 and 2025. Seven hundred and eighty-four animals, representing over 58 species (including primates, marsupials, rodents, and felids), were vaccinated without any adverse events reported. **Results**: Retrospective mortality data from 20 participating zoological institutions revealed an overall 96.7% reduction—and, in many cases, a complete elimination—of toxoplasmosis-associated deaths post vaccination. **Conclusions**: These results demonstrate, for the first time, consistent and broad-spectrum protection against *T. gondii* of different strains in a wide array of captive wildlife species. This universal vaccine represents a promising tool for toxoplasmosis prevention in zoological collections, with significant implications for animal health and conservation strategies.

## 1. Introduction

*Toxoplasma gondii* is a widespread protozoan parasite and one of the most common agents of zoonotic infections in humans [1]. The parasite can infect nearly all warm-blooded species as intermediate hosts, but its sexual reproduction phase occurs exclusively in felids, its ultimate host. Although some species—such as Old World primates, rats, cattle, and horses—exhibit relative resistance to the parasite, others—such as macropods [2] and other marsupials, ring-tailed lemurs (*Lemur catta*) [3], squirrel monkeys (*Saimiri* spp.) [4,5], meerkats (*Suricata suricatta*) [6], and Pallas’ cats (*Otocolobus manul*) [7,8]—are highly susceptible. This extreme vulnerability is believed to be the result of divergent evolutionary histories, leading to immune systems poorly adapted to resist *T. gondii* infection [9]. Infected animals commonly present neurological symptoms, lethargy, respiratory distress, appetite loss, and even sudden death. Treatment typically involves the administration of sulfonamides, diclazuril, or clindamycin [7,10], but these therapies are often ineffective in preventing death, underscoring the importance of preventive strategies.

This issue is critical in zoos and wildlife reserves where outbreaks of toxoplasmosis pose a serious threat to conservation efforts for endangered species. Among the risk factors for *T. gondii* infections, open enclosures and contact with reservoirs—cats and rodents—are commonly identified [11]. The occurrence of outbreaks is also often correlated with flooding or abundant rainfall in zoos, which facilitates the transmission of *T. gondii* oocysts via water, making its spread unpredictable and hard to prevent. Toxoplasmosis-related mortality has been widely documented in this species in various studies, particularly in New World primates and marsupials [9,12,13]. Squirrel monkeys are considered the most vulnerable to *T. gondii* infection, and can die suddenly without exhibiting clear signs of the disease [12,14]. Similarly, *L. catta* [3,9,15], wallabies [2,16,17], kangaroos [2,16,18,19], and meerkats [6,9,20] continue to experience high levels of morbidity and mortality from the parasite in captive settings. The susceptibility of the species may be linked to their life habits. A comparative study of host susceptibility in 14 primate species revealed that all of the 10 individuals of Old World primate species tested were seropositive for toxoplasmosis, but none succumbed to the infection [21]. In contrast, only 6% of the 37 tested neotropical primates were positive for anti-*T. gondii* antibodies, and toxoplasmosis-related deaths were reported within this group, including two Black bearded saki (*Chiropotes satanas*), Owl monkeys (*Aotus trivirgatus*), two Golden lion tamarins (*Leontopithecus rosalia*), and Black-fronted titi monkey (*Callicebus nigrifrons*) between 2013 and 2019. Interestingly, the 6% of seropositive animals included Tufted capuchins (*Sapajus apella*), which exhibited an increased resistance to *T. gondii*: Three out of six individuals of this species that were tested were seropositive, yet none died of the infection, indicating an increased resistance to *T. gondii*. The authors suggested that Tufted capuchins, having evolved to forage for food on the ground and thus being exposed to the parasite more often than the other neotropical primate species, developed an effective immune response against *T. gondii*. This could explain the heightened vulnerability of squirrel monkeys which spend their entire life in the treetops. The particular susceptibility of New World monkeys has been frequently reported [22]. Some researchers propose that the vulnerability of Australian marsupials and lemurs from Madagascar stems from their evolutionary isolation from the parasite, as there are no native felid species in Australia and Madagascar [23]. Since both acute and chronic forms of toxoplasmosis can lead to death in vulnerable mammal species [13], effective preventive strategies are urgently needed. Some zoos, such as Africam Safari, have ceased keeping certain species due to the challenges in preventing toxoplasmosis-related mortality. Preventive measures usually include restricting cat access to enclosures, thorough washing of fruits and vegetables, and pre-feeding meat inspections. However, despite those preventive measures, toxoplasmosis remains an obstacle for the preservation of captive wildlife.

To date, no market-approved vaccine exists for toxoplasmosis in either humans or captive animals, except for sheep. The only vaccine available—Ovilis^®^ Toxovax (MSD Animal Health)—is based on a live attenuated *T. gondii* parasite, and has been approved for toxoplasmosis prevention in sheep, but is not recommended for high-risk animals. Using a live attenuated vaccine has several limitations, including low stability, biohazard considerations, and the possibility of the reversion of the attenuated phenotype [24]. Vaccines based on inactivated pathogens do not pose a risk of inducing disease and offer a safe option of disease prevention in vulnerable species. An intranasal vaccine composed of maltodextrin nanoparticles conjugated with antigens of inactivated *T. gondii*, known as VXN-Toxo, has shown promising results in various experimental settings. These nanoparticles are inert and non-toxic, they do not cross the blood–brain or nose–brain barriers, do not induce inflammation, and are fully eliminated via the gastrointestinal tract [25]. Most importantly, they facilitate the passage of the antigen through the mucosal membranes and prolong its residence time in the nasal cavity, therefore increasing the stimulation of mucosal immune responses. The successful use of this vaccine has been reported in mice [26], sheep [27], and non-human primates [28]. Studies in *S. boliviensis* demonstrated that VXN-Toxo elicits a strong Th1-polarized immune response without triggering anti-*T. gondii* antibody production [28].

Between 2017 and 2025, the VXN-Toxo vaccine was used in vaccination campaigns in 29 zoos in various European (France, Belgium, The Netherlands, Hungary, Isle of Man, Portugal, Greece, and Denmark) and American (Colombia, Brazil, and Mexico) countries. This publication presents a retrospective overview of the impact of the 2017–2025 VXN-Toxo vaccination campaign on toxoplasmosis-associated mortality in zoo animals from 20 of those institutions, shining a light on the marked decrease in the number of deaths after the vaccination campaign.

## 2. Materials and Methods

### 2.1. The Ethics Statement

The experimental VXN-Toxo vaccine used in this study has not been approved for marketing in the European Union or the Americas. However, in accordance with Article 112 of Regulation (EU) 2019/6 [29], the administration of the vaccine was permitted under the veterinary cascade, which allows the use of unauthorized veterinary medicinal products in cases where no authorized alternative is available for a given condition and species. In the absence of a licensed vaccine against *T. gondii* for the target wildlife species held in zoological institutions, the decision to use the experimental product under the last step of the cascade was made under the direct professional responsibility of the attending veterinarian, who evaluated the risks and potential benefits, and ensured compliance with applicable legal and ethical standards.

This approach is further supported by guidance from the Federation of Veterinarians of Europe (FVE), which recognizes that the cascade system permits veterinarians to prescribe non-authorized medicinal products when justified and when no authorized alternatives exist, provided such use is ethically sound and professionally documented, as described in 2019/6/EU of the European Parliament and of the Council of 11 December 2018 on veterinary medicinal products [29]. Formal agreement for the first use of the vaccine in squirrel monkeys was obtained from the European studbook manager at the European Association of Zoos and Aquaria (EAZA) by Bioparc Doué-la-Fontaine.

Each zoo had responsibility for the animals and was not subject to Directive 2010/63/EU of the European Parliament and of the Council of 22 September 2010 on the protection of animals used for scientific purposes [30]. All animals involved in the study were monitored daily by zookeepers, and each institution’s husbandry and veterinary teams were responsible for their well-being, health, and medical care.

### 2.2. VXN-Toxo Vaccine Preparation

The VXN-Toxo vaccine was prepared as previously described [28] and consists of purified, inactivated *Toxoplasma gondii* tachyzoites conjugated to lipid core maltodextrin nanoparticles (NPLs), produced under Good Manufacturing Processes (GMP), as described earlier [28]. The final formulation was packaged in multi-dose vials.

### 2.3. Vaccinations

**Figure 1:**
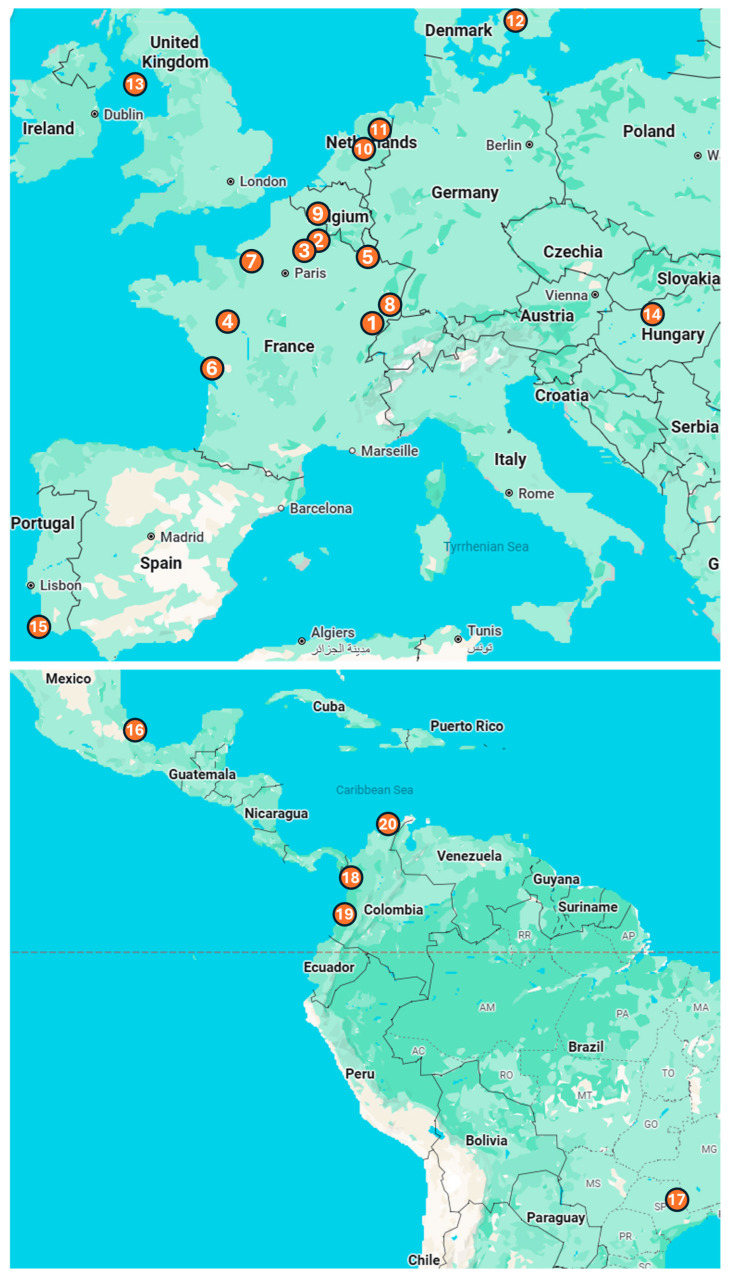
Location of the 20 zoos that participated in Vaxinano’s vaccination campaigns. Europe: (1) Besançon Zoo, (2) Parc d’Isle, (3) Exotic Garden, Foster’s Center for Small Animals, (4) Bioparc of Doué-la-Fontaine, (5) Amnéville Zoo, (6) La Palmyre Zoo, (7) Biotropica Zoo, (8) Zoo and Botanical Garden of Mulhouse, (9) Pairi Daiza Zoo, (10) Apenheul Zoo, (11) Wildlands, (12) Copenhagen Zoo, (13) Curraghs Wildlife Park, (14) Budapest Zoo and Botanical Garden, (15) Lagos Zoo. Central and South America: (16) Africam Safari Zoo, (17) Quinzinho de Barros Zoo, (18) Parque de la Conservacion, (19) Cali Zoo, (20) Baranquilla Zoo.

The vaccination campaigns took place in 29 European and American zoos, among which 20 shared their data on mortality before and after the vaccination (Figure 1).

### 2.4. Safety of VXN-Toxo in Captive Wildlife

Each intranasal administration of VXN-Toxo was accompanied by a mandatory detailed adverse effect form completed by the attending veterinarian at each participating zoo. The animals were monitored according to the usual husbandry and veterinary routines of each institution. Adverse effects were defined as respiratory or ocular signs (sneezing, epistaxis, nasal discharge, epiphora) or behavioral or pain-related responses including anxiety, aggression, impaired or reduced mobility, anorexia, self-mutilation, abnormal posture or activity, social withdrawal, and unresponsiveness.

### 2.5. Immunization

The *T. gondii* infection status was assessed before beginning the vaccination program in some animals using an immunochromatographic IgG-IgM test (Toxoplasma ICT IgG-IgM, LDBio, Lyon, France). All animals were immunized intranasally at least three times (Prime: D0, 1st Booster: D0 + 1 mo; 2nd Booster: D0 + 5–12 mo) into a single nostril with a nasal spray device (VP7-50 232 NE pump, Aptar Pharma, Le Vaudreuil, France). Vaccination was repeated into the other nostril if the animal sneezed or blew the vaccine out of its nose. Vaccination was performed on either conscious or anesthetized animals, depending on the decision of the attending veterinarians from each participating zoo.

### 2.6. Toxoplasma-Related Mortality in Zoos

Mortality data was collected from 20 out of the 29 zoos where vaccination campaigns were implemented. Veterinarians from those zoos shared reports of diagnosed toxoplasmosis mortality cases between 2007 and 2025, including the name of the species affected, the yearly number of animals of this species that died due to toxoplasmosis, the total number of animals of this species housed this particular year, and the diagnostic method used to confirm toxoplasmosis as the cause of death.

The second part of this study comprised an online survey composed of the following questions, with the following response options:

1. Did you record any toxoplasmosis-related deaths before the vaccination was introduced? (Yes/No)
2. Is a specific diagnosis systematically made when a deceased animal is suspected of having toxoplasmosis? (Yes/No/Not applicable)
3. If yes, at what moment was the diagnosis carried out? (When the symptoms were detected/Post-mortem/Not applicable)
4. What was the diagnostic method used? (Open question)
5. If no, is it possible that any of the undiagnosed animals died from toxoplasmosis, given the clinical signs? (Yes/No/Not applicable)
6. Have you implemented any measures other than vaccination to prevent toxoplasmosis? (Yes/No/Not applicable)
7. If yes, what measures? (Open question)
8. What prompted you to set up a vaccination campaign with VXN-Toxo? (Open question)
9. Would you recommend the vaccination to other zoos? (Yes/No/Not applicable)

The responses were collected, analyzed, and summarized.

### 2.7. Toxoplasmosis Diagnosis

Several zoos, such as La Palmyre, Parc d’Isle, Bioparc of Doué-La-Fontaine, Amnéville Zoo, Botanical Garden of Mulhouse, Apenheul, Wildlands, Copenhagen Zoo, Lagos Zoo, Africam Safari Zoo, Budapest Zoo and Botanical Garden, Quinzinho de Barros Zoo, Cali Zoo, and Baranquilla Zoo confirmed toxoplasmosis through necropsy, the examination of organ lesions, histopathology, and/or immunohistochemistry (IHC). Some establishments, like Curraghs Wildlife Park and Pairi Daiza, based the diagnosis on the physical examination and disease symptoms. PCR diagnosis at the time of symptoms was used by Budapest Zoo and Botanical Garden, Lagos Zoo, and Quinzinho de Barros Zoo. Besançon Zoo and Biotropica Zoo did not perform specific diagnostic procedures.

## 3. Results

### 3.1. Vaccinated Animals

**Figure 2:**
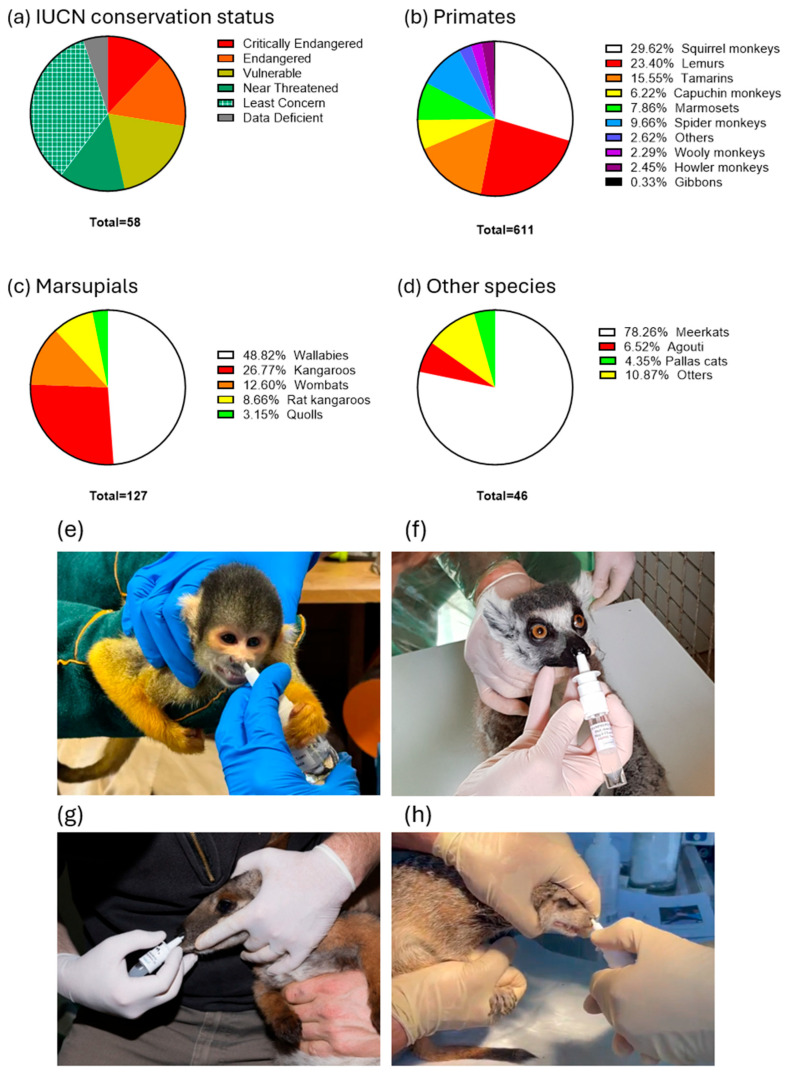
(**a**) International Union for Conservation of Nature (IUCN) conservation status in the vaccinated species as proportions of the study sample. Taxa among the total number of primates (**b**), marsupials (**c**), other species (**d**) vaccinated with VXN-Toxo, as proportions of the study sample. Intranasal vaccinations of *Saimiri* spp. from Mulhouse Zoo (**e**), *L. catta* from Champrepus Zoo (**f**), a *P. xanthopus* from Mulhouse Zoo (**g**), a *S. suricatta* from Attica Park in Greece (**h**).

Due to the significant mortality caused by toxoplasmosis reported prior to the introduction of the vaccination, many zoos across Europe and the Americas (Figure 1) initiated vaccination campaigns. A total of 611 primates of 42 species, 127 marsupials of 12 species, and 46 other animals, including 2 Pallas’ cats, 3 Azaras agoutis (*Dasyprocta azarae*), 36 meerkats, and 5 Asian small-clawed otters (*Aonyx cinereus*), were vaccinated with VXN-Toxo (Table 1, Figure 1 and Figure 2). Of the 58 species vaccinated, 35 (60%) are listed as threatened or at risk—categorized as Critically Endangered, Endangered, Near Threatened, or Vulnerable—according to the IUCN Red List of Threatened Species. The analysis of adverse event reports revealed no vaccine-associated side effects in vaccinated animals, indicating excellent vaccine tolerance.

### 3.2. Toxoplasmosis-Related Mortality in Captive Animals

**Figure 3:**
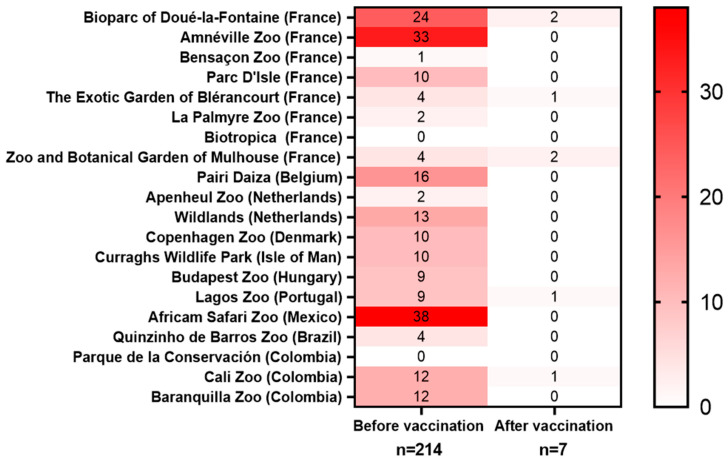
Heatmap of toxoplasmosis-related deaths before and after the vaccination. Data collected from 20 zoos. Animals that were seropositive to *T. gondii* before vaccination were excluded from the data pool.

Twenty zoos shared their data on the toxoplasmosis-associated mortality they experienced before and after the vaccination campaigns (Table 2, Figure 3). Thirteen of them also participated in an online survey, sharing information on their local implementation of toxoplasmosis prevention strategies.

#### 3.2.1. Bioparc of Doué-la-Fontaine (France, Doué-la-Fontaine)

Before vaccination: Significant mortality due to toxoplasmosis occurred at Bioparc de Doué-la-Fontaine. One *L. catta* died in 2005, and two out of fourteen *S. boliviensis* died in 2007. Starting in 2014, repeated outbreaks were observed in *S. boliviensis*: six out of thirty-one died in 2014, eight out of fifteen in 2015, two out of nineteen in 2016, and five out of fifteen in 2017. Systematic diagnosis was performed through necropsy and histopathology, and the diagnosis was sometimes confirmed via immunohistochemistry.

After vaccination: Bioparc was the first zoo to test the vaccine, beginning in late 2017. Seventeen *S. boliviensis* received three to five doses of the vaccine between 2017 and 2020, including two animals that were seropositive for *T. gondii*. Four toxoplasmosis-related deaths were documented in the years following the vaccinations: two previously described seropositive *S. boliviensis*, and two others that were seronegative at the time of their first vaccinations. The seronegative animals died approximately two months after their second dose (Section 3.3), and the seropositive ones in 2018 and 2020 each. It is worth mentioning that those animals were vaccinated intranasally using a pipette, an initial iteration of the vaccine administration method later deemed inefficient. Since switching to a spray-based administration method, no further deaths from toxoplasmosis have been reported at the zoo, except for one unvaccinated *Procavia capensis* that died in 2024, with the diagnosis confirmed by necropsy and histopathology.

#### 3.2.2. Amnéville Zoo (France, Amnéville)

Before vaccination: Amnéville Zoo experienced one of the most severe toxoplasmosis-related mortality events in *S. boliviensis*, with thirty-three deaths recorded before vaccinations began. The first outbreak occurred in 2010, with three Squirrel monkeys dying, and another death followed in 2011. The cases involved sudden deaths, neurological symptoms such as eyelid paralysis and dyspnea, and animals dying shortly after initial symptom onset. In 2012, five more monkeys died suddenly, followed by nine deaths in 2013, eight in 2014, seven in 2016, and one in 2017. Diagnoses were confirmed at least by the observation of clinical signs and histopathological evaluation, and, in several cases, by immunohistochemistry or PCR, which identified the *T. gondii* type II strain. Two of the deaths mentioned were miscarriages attributed to toxoplasmosis.

After vaccination: Between 2017 and 2019, nine *S. boliviensis* received five doses of the vaccine, and between 2019 and 2020, eighteen *N. rufogriseus* received three doses. Only one vaccinated *S. boliviensis* developed toxoplasmosis. The animal was *T. gondii* seropositive before the vaccination, and started showing weight loss, balance issues, and breathing difficulties five months after the fourth dose of the vaccine. Interestingly, the animal recovered fully after treatment with sulfamethoxazole, trimethoprim, and clindamycin—unlike most infected squirrel monkeys, which typically die within two to three days. The monkey later received a fifth dose and was transferred to Lagos Zoo in Portugal, where it died from an unrelated cause. One vaccinated seropositive *N. rufogriseus* died in 2021, a year after the third vaccine dose, showing neurological symptoms and nasal discharge. The diagnosis on those two animals was based on symptoms at the time of death and histopathology.

#### 3.2.3. Besançon Zoo (France, Besançon)

Before vaccination: In 2010, Besançon Zoo recorded the death of 1 out of 35 housed rock wallabies (*Petrogale* spp.). The cause of death was confirmed during necropsy. No other toxoplasmosis-related deaths were reported before vaccination began.

After vaccination: Vaccinations with VXN-Toxo began in 2018. Between 2018 and 2021, eleven *S. boliviensis* received between three and five doses of the vaccine. No cases of toxoplasmosis were documented since then.

#### 3.2.4. Parc d’Isle (France, Saint-Quentin)

Before vaccination: Parc d’Isle Zoo experienced its first toxoplasmosis outbreak in 2023. Three out of fourteen unvaccinated *L. catta* and one out of ten *N. rufogriseus* died from toxoplasmosis. The diagnosis was confirmed by serology and PCR when symptoms appeared, and later by histopathology and IHC after death. No toxoplasmosis-related deaths had been reported at the zoo before this outbreak.

After vaccination: Vaccination began during the 2023 outbreak. Between 2023 and 2024, six *Saimiri* spp., thirteen *L. catta*, nine *N. rufogriseus*, and four *S. suricatta* received three doses of the VXN-Toxo vaccine. One *L. catta* had died of toxoplasmosis four days before vaccinations began and was therefore not vaccinated. Among the thirteen vaccinated lemurs, two already showed signs of acute toxoplasmosis at the time of their first dose and died three and four days later. Another seronegative *L. catta* became infected between the first and second vaccine doses, developed symptoms shortly after the first booster, and was diagnosed with toxoplasmosis. The animal was successfully treated with clindamycin and metronidazole and fully recovered within three weeks. It later received the third vaccine dose in January 2024, at which point serology confirmed the presence of *T. gondii* antibodies, and hematological and biochemical blood parameters had returned within reference levels for the species.

#### 3.2.5. The Exotic Garden, Foster’s Center for Small Primates (France, Blérancourt)

Before vaccination: Two *L. catta* individuals died from toxoplasmosis in 2023 and 2024. Additionally, one unvaccinated *N. rufogriseus* died in 2025. These cases occurred before the vaccination campaign or in animals not included in the campaign.

After vaccination: Between 2024 and 2025, the following animals received three doses of the vaccine: twelve *E. fulvus*, six *C. jacchus*, five *L. catta*, four *S. imperator*, four *C. pygmaea*, three *C. goeldii*, three *S. midas*, two *E. macaco*, two *S. oedipus*, two *Saimiri* spp., and one *V. variegata variegata*. One vaccinated *L. catta* died of toxoplasmosis eleven days after receiving the second dose (Section 3.3).

#### 3.2.6. La Palmyre Zoo (France, Les Mathes)

Before vaccination: La Palmyre Zoo recorded two deaths due to toxoplasmosis in meerkats—one in 2013 and another in 2016. Both cases were confirmed through histopathological analysis.

After vaccination: Preventive vaccination began in 2022 in squirrel monkeys, but not meerkats. Between 2022 and 2023, six *S. sciureus* received three doses of the vaccine. No further toxoplasmosis-related deaths were reported following the start of the vaccination program.

#### 3.2.7. Biotropica Zoo (France, Val-de-Reuil)

Biotropica Zoo observed deaths in unvaccinated wallabies and kangaroos after the vaccination campaign started. Between 2023 and 2024, twenty-one *S. boliviensis* received three doses of the VXN-Toxo vaccine. The zoo now applies yearly booster doses for adult squirrel monkeys and a full three-dose schedule for young ones.

In 2024, two *N. parma*, and one *M. fuliginosus* confirmed toxoplasmosis deaths, and one suspected toxoplasmosis death in *N. parma* was documented. No systematic diagnostic tests were carried out, but protozoan infection was suspected based on clinical signs. In one wallaby, a brain sample tested positive for a protozoan infection via PCR. The vaccination of wallabies is currently under consideration. No toxoplasmosis-related deaths in vaccinated animals were reported following the start of the vaccination.

#### 3.2.8. Zoo and Botanical Garden of Mulhouse (France, Mulhouse)

Before vaccination: In 2017, four out of eight *S. boliviensis* died at the Zoo and Botanical Garden of Mulhouse. These deaths were confirmed as toxoplasmosis cases through histopathological analysis.

After vaccination: Between 2017 and 2019, fourteen *P. xanthopus*, five *S. boliviensis*, and two *O. manul* received from three to five doses of the vaccine. Despite full vaccination, one *S. boliviensis* died in 2022 and another in 2023—both had received five doses (Section 3.3). Diagnosis in these cases was also confirmed by histopathology.

#### 3.2.9. Pairi Daiza Zoo (Belgium, Brugelette)

Before vaccination: In 2016, a toxoplasmosis outbreak at Pairi Daiza Zoo caused the death of sixteen *S. boliviensis peruviensis*. The diagnosis was based on physical examination.

After vaccination: Between 2021 and 2023, thirty-two *S. boliviensis* received from three to five doses of the vaccine. The vaccination campaign also included twenty-five *L. catta*, seven *S. oedipus*, six *P. pithecia*, five *V. variegata variegata*, and two *E. coronatus*. Another outbreak occurred in 2024, but no vaccinated *S. boliviensis* were affected. The only two deaths from toxoplasmosis occurred in unvaccinated *L. catta*.

#### 3.2.10. Apenheul Zoo (The Netherlands, Apeldoorn)

Before vaccination: At Apenheul Zoo in The Netherlands, two *A. seniculus* (Venezuelan red howler monkeys) died from toxoplasmosis—one in 2021 and another in 2022. The first case was confirmed by histopathology, and the second by immunohistochemistry.

After vaccination: In 2023, the zoo vaccinated four *A. seniculus*, two *P. pithecia*, two *L. chrysomelas*, and one *S. bicolor*. No further toxoplasmosis-related deaths were reported after the start of the vaccination campaign.

#### 3.2.11. Wildlands (The Netherlands, Emmen)

Before vaccination: In 2012, a toxoplasmosis outbreak at Wildlands Zoo led to the death of twelve out of forty-four *L. catta*. The diagnosis was confirmed by histopathology. Another death due to toxoplasmosis was recorded in a *S. boliviensis* in 2019.

After vaccination: The vaccinations began in 2022. A total of twenty-one *S. boliviensis*, fifteen *L. catta*, five *W. bicolor*, four *C. pygmaea*, and two *V. variegata variegata* received two out of three planned doses of the vaccine. No further deaths were reported following the start of the vaccination campaign.

#### 3.2.12. Copenhagen Zoo (Denmark, Copenhagen)

Before vaccination: Between 2014 and 2022, toxoplasmosis-related deaths were observed across multiple species at Copenhagen Zoo. Among *L. catta*, one out of six died in 2014, two out of six in 2015, and one in five in 2019. In *M. giganteus*, one death in a group of nine was documented in 2015 and another in one of nine in 2019. In meerkats, one of four died in 2019, and one of nine in 2022. Additional deaths were recorded in other species: one in six Tasmanian wombats (*V. ursinus*) in 2018, one of two Arctic foxes (*Vulpes lagopus*) in 2020, and one of three muskoxen (*Ovibos moschatus*) in 2021. Diagnosis was confirmed by histopathology and immunohistochemistry in at least one *L. catta*.

After vaccination: From 2021 to 2024, the zoo vaccinated thirteen *V. ursinus*, eleven *L.catta*, four *P. xanthopus*, four *D. viverrinus*, three *B. penicillata*, and two *P. tridactylus*. The zoo also plans to vaccinate additional animals, including newly arrived *L.catta*, two more *V. ursinus*, four *D. viverrinus*, one *P. xanthopus*, one *P. tridactylus*, and one *B. penicillata*. No further deaths were reported following the start of the vaccination campaign.

#### 3.2.13. Curraghs Wildlife Park (Isle of Man)

Before vaccination: A major toxoplasmosis outbreak occurred in 2018 among Asian small-clawed otters (*A. cinereus*), resulting in eight deaths out of fourteen animals. Diagnosis was based on clinical symptoms and the detection of tachyzoites during necropsy. The outbreak was suspected to be linked to a stress event, specifically, anesthesia for medical procedures. Additionally, one *S. suricatta* died of toxoplasmosis in 2017, and one *S. boliviensis peruviensis* in 2020.

After vaccination: The vaccinations began in 2023. At least five *A. cinereus* were vaccinated. In total, eleven meerkats, nine *L. catta*, *seven S. boliviensis boliviensis*, three *D. azarae*, two *A. fusciceps robustus*, two *H. alaotrensis*, two *V. variegata rubra*, and two *V. variegata variegata* received two doses of the vaccine. No further deaths were reported following the start of the vaccination campaign.

#### 3.2.14. Budapest Zoo and Botanical Garden (Hungary, Budapest)

Before vaccination: Budapest Zoo recorded several toxoplasmosis-related deaths. In Tammar wallabies (*N. eugenii*), one of nine died in 2013, two of five in 2015, one of four in 2016, and one of three in 2018. In Bennett’s tree-kangaroos (*D. bennettianus*), one of twenty-five died in 2015, and two of eleven in 2017. One dwarf goat (*Capra aegagrus hircus*) also died in 2018. Diagnosis in all cases was confirmed by immunohistochemistry and PCR.

After vaccination: Between February and March 2023, the zoo vaccinated sixteen *D. bennettianus*, six *B. gaimardi*, four *M. fuliginosus*, three *V. ursinus*, and two *N. parma*, each receiving two doses of the vaccine. No further toxoplasmosis-related deaths have been reported since vaccination.

#### 3.2.15. Lagos Zoo (Portugal, Barão de São João)

Before vaccination: Lagos Zoo reported several toxoplasmosis-related deaths in *S. boliviensis* and *L. catta*. In 2018, two confirmed and one suspected case occurred in *S. boliviensis*. In 2019, there was one confirmed and one suspected case. Additional confirmed cases were reported in 2020 and 2022. In 2020, three of ten *L. catta* died. Diagnosis was confirmed by PCR during clinical symptoms and by histopathology and immunohistochemistry after death.

After vaccination: The vaccinations began in 2021. The following animals received vaccine doses: six *L. catta*, eight *C. geoffroyi*, five *S. suricatta*, three *V. variegata rubra*, three *S. boliviensis*, two *C. pygmaea*, two *S. imperator*, two *S. midas*, and two *S. oedipus*. In 2025, additional vaccines were administered to ten more *L. catta*, two *V. variegata variegata*, four *C. pygmaea*, twenty-three *S. boliviensis*, and four *W. bicolor*. One of the *S. boliviensis*, which had received two doses of the vaccine in the 2021 campaign, died in 2022 (Section 3.3).

#### 3.2.16. Africam Safari Zoo (Mexico, Puebla)

Before vaccination: Africam Safari Zoo experienced severe toxoplasmosis-related mortality, particularly in Australian marsupials. As a result, the zoo stopped housing these species starting in 2022. A major outbreak in 2013 led to the deaths of fourteen of twenty-one *N. rufogriseus*, six of eight *O. rufus*, two of thirteen *S. suricatta*, four of six *S. sciureus*, and one out of twenty-four *L. catta*.

Frequent deaths in *N. rufogriseus* were also recorded between 2012 and 2017: one in 2012, fourteen in 2013, three in 2014, one in 2015, and one in 2017. Among *L. catta*, one death occurred in 2013, followed by three deaths in a group of forty-two animals in 2019 and one death in a group of forty in 2021. One additional *O. rufus* died in 2015.

Diagnosis was primarily based on lesions seen during necropsy and the identification of apicomplexan parasite structures in lung smears stained with Romanowsky-type stain. Immunohistochemistry and histopathology were used later to assess the systemic extent of infection.

After vaccination: The zoo started the preventive vaccination campaign in 2025; fourteen *O. rufus* and five *S. suricatta* received two doses of the vaccine as of June 2025.

#### 3.2.17. Quinzinho de Barros Zoo (Brazil, Sorocaba)

Before vaccination: Quinzinho de Barros Zoo in Brazil recorded two deaths due to toxoplasmosis in Southern muriquis (*B. arachnoides*) with one death in four animals in 2021 and another in three animals in 2022. In 2023, one *L. catta* also died in a group of four. The diagnosis was confirmed by PCR in the muriqui cases and by immunohistochemistry in the lemur. These deaths in primates prompted the zoo to begin a vaccination program.

After vaccination: The vaccinations began in 2024. The zoo vaccinated a wide range of primates, including twenty *S. bicolor*, five *A. caraya*, four *L. chrysomelas*, four *A. marginatus*, three *L. catta*, three *S. midas*, three *L. fuscicollis*, three *L. cana*, three *B. arachnoides*, two *P. vieirai*, two *A. chamek*, two *A. trivirgatus*, two *L. rosalia*, one *A. trivirgatus*, and one *S. niger*. Each received two to three doses of the vaccine. No deaths have been reported in vaccinated individuals to date.

#### 3.2.18. Parque de la Conservación (Medellín—Colombia)

Before vaccination: No toxoplasmosis-related deaths were reported in Parque de la Conservación in Medellín, Colombia, prior to the implementation of the vaccination campaign.

After vaccination: Vaccinations began in 2022. The zoo immunized twenty-one *A. hybridus*, eleven *C. albifrons*, nine *S. sciureus*, eight *A. fusciceps*, six *A. seniculus*, four *S. geoffroyi*, four *C. capucinus*, three *S. apella*, five *S. oedipus*, one *C. pygmaea*, and one *L. lagothricha*. No toxoplasmosis-related deaths have been reported since the start of the vaccination program.

#### 3.2.19. Cali Zoo (Colombia, Santiago de Cali)

Before vaccination: The Zoo of Cali in Colombia recorded toxoplasmosis-related deaths between 2020 and 2024. In 2020, one *A. lemurinus*, one *L. catta*, one *C. pygmaea*, one *S. oedipus*, two *S. sciureus*, and two *O. leucopus* died, with diagnosis confirmed by histopathology and PCR. One *N. rufogriseus* died in 2021. In 2023, additional deaths included one *A. lemurinus*, one *L. lagothricha*, one *V. variegata variegata*, and one *N. rufogriseus*. In 2024, three more *N. rufogriseus* died; one of them had been vaccinated, but vaccine administration failure was suspected. Diagnosis in 2023 also included the direct observation of tachyzoites via the Wright–Giemsa staining of tracheal cytology in *N. rufogriseus* and *L. lagothricha*.

After vaccination: The vaccinations were administered between 2022 and 2023. The vaccinated animals included eleven *S. suricatta*, nine *S. sciureus*, eight *S. leucopus*, seven *L. catta*, seven *L. lagothricha*, seven *C. pygmaea*, seven *S. apella*, six *N. rufogriseus*, five *L. fuscicollis*, four *C. capucinus*, four *V. variegata variegata*, two *A. fusciceps*, and two *H. lar*. Despite these efforts, a suspected administration failure was linked to a toxoplasmosis-related death in one of the vaccinated wallabies in 2024.

#### 3.2.20. Baranquilla Zoo (Colombia, Baranquilla)

Before vaccination: Baranquilla Zoo in Colombia suspected the deaths of two *N. rufogriseus* between 2007 and 2008, but the diagnosis was not confirmed by histopathology. In 2018, the deaths of one *L. catta* and one *V. variegata variegata* were confirmed. One more *L. catta* and two *S. geoffroyi* died the next year. In 2020, one *A. lemurinus*, one *S. geoffroyi*, and one *S. suricatta* died, and the death of one *S. oedipus* was documented in 2021. The last death documented occurred in 2022—one *S. suricatta* died. The diagnosis was confirmed by histopathology in all the death cases between 2018 and 2020.

After vaccination: In 2022, the following animals were vaccinated: eleven *A. fusciceps*, nine *C. pygmaea*, eight *A. hybridus*, nine *S. apella*, seven *S. geoffroy*, four *S. oedipus*, three *A. lemurinus*, two *L. fuscicollis*, two *S. leucopus*, and one *A. belzebuth*. No toxoplasmosis-related deaths were documented ever since, as described in the recent publication [13].

### 3.3. Summary of Toxoplasmosis-Associated Mortality and Vaccinations

**Mortality before the vaccination**: A total mortality of 214 animals was reported in 20 participating zoos before the start of the vaccination campaigns (Figure 3, Table 2). Before the start of the vaccination campaign, the highest mortality rates were reported in *L. catta, Saimiri* spp., meerkats, wallabies, and kangaroos—findings that are consistent with previously published data. Among the deaths reported before the vaccinations started, the most represented species were *Saimiri* spp. (41.5%), wallabies (*N. rufogriseus*, *N. parma*, or *N. eugenii*, 18.2%), *L. catta* (15.9%), kangaroos (*O. rufus*, *M. fuliginosus*, or *M. giganteus*, 4.2%), and *S. suricatta* (3.7%). Those species have been previously described as particularly vulnerable to toxoplasmosis [16]. Consequently, the animals that were the most impacted by toxoplasmosis were also the ones that were the most frequently vaccinated with VXN-Toxo: *Saimiri* spp. (23.1%), *L. catta* (13.3%), wallabies (7.9%), meerkats (4.6%), and kangaroos (4.3%). An analysis of the adverse event reports revealed no vaccine-associated side effects among 784 vaccinated animals representing 58 different species, indicating the excellent tolerability of the vaccine.

**Mortality after the vaccination**: The vaccination campaign was followed by a marked reduction in toxoplasmosis-related mortality. In contrast to the more than 214 deaths reported before the vaccinations, only 23 deaths were reported in the years following the vaccinations, out of which 10 occurred in animals that were unvaccinated, 5 in animals that were vaccinated while being *T. gondii*-seropositive or even showing toxoplasmosis symptoms, 1 occurred in a *S. boliviensis* of unknown status of vaccination, and only 7 occurred in *T. gondii*-naïve animals that were vaccinated (Figure 3). Of those seven animals, vaccine administration failure was suspected in four of them. Additionally, two of them died 3–4 years after receiving the last booster, one became infected before completing the whole course or vaccinations, and in two a bacterial co-infection was detected at the time of autopsy. When assessing the vaccine efficacy based on the number of deaths in vaccinated seronegative animals, the vaccination decreased the mortality by 96.7%.

The first two deaths in vaccinated seronegative animals occurred in Bioparc of Doué-la-Fontaine, where vaccine administration failure was suspected during the first immunizations. The zoo was the first establishment in which the vaccinations were implemented, and the administration route had not yet been optimized at the time of the first immunizations. The first two doses in squirrel monkeys were administered via the intranasal route by a pipette tip and without anesthesia, and the animals were sneezing and blowing the vaccine out of their noses. The failure of vaccine administration in at least two of the animals was presumptively confirmed by the toxoplasmosis-associated death two months after receiving the second dose of the vaccine. This mortality prompted the zoo to seek out subsequent booster doses—the third dose was administered via subcutaneous injection, and the fourth one by intranasal instillation in anesthetized animals. No mortality in vaccinated seronegative animals was reported in Bioparc since the administration of the third vaccine dose in the remaining thirteen *S. boliviensis*.

The third animal, an *L. catta* from The Exotic Garden, Foster’s Center for Small Primates of Blérancourt, died before completing the whole course of the three-vaccination regimen. The lemur tested seronegative for *T. gondii* IgG-IgM antibodies in the blood before receiving the first dose and at the time of death. It died eleven days after receiving the second dose, having developed hyperthermia and neurological symptoms five days earlier. *Pseudomonas aeruginosa* was detected in the lungs and brain, and the *T. gondii* DNA was detected via PCR on tissue samples. However, the necropsy did not show *T. gondii* cysts or other signs of the presence of the parasite, suggesting that toxoplasmosis might have been unrelated to the clinical outcome.

The fourth and fifth deaths occurred at the Zoo and Botanical Garden of Mulhouse. Two deaths in vaccinated *S. boliviensis* were recorded: one in 2022, and another in 2023. Both animals had received five doses of the vaccine between 2017 and 2019, so three and four years before their death, respectively.

The sixth case of death in vaccinated animals was a *S. boliviensis* that died at Lagos Zoo, and the time between the second dose and death was approximately nine months. The necropsy revealed severe lesions in the liver, meningeal congestion, and microhemorrhages in the encephalon. Histopathological examination identified protozoan cysts in the thalamus, and systemic *T. gondii* infection was confirmed by immunohistochemistry and PCR-based methods, with high parasite burdens in the liver and lung tissues. Although *E. coli* was isolated from blood and viscera, its presence was considered incidental or secondary, with no evidence indicating a primary role in the pathogenesis. Vaccine administration at Lagos Zoo was performed without anesthesia, which does not allow the exclusion of administration failure due to sneezing out the vaccine during administration.

The seventh death occurred at Cali Zoo—one of the vaccinated Red-necked wallabies died in 2024. The veterinarians suspect a failure of vaccine administration in this individual.

### 3.4. Toxoplasma Prevention Survey

Of the 20 zoological institutions that submitted mortality and/or vaccination data for this study, 13 also responded to a follow-up survey regarding toxoplasmosis prevention strategies. All respondents reported confirmed cases of toxoplasmosis-related mortality prior to the implementation of vaccination, and 12 of 13 indicated that a systematic diagnostic approach is employed whenever toxoplasmosis is suspected as the cause of death. The majority (9/12) reported conducting post mortem examinations through necropsy and organ analysis. Histological and/or immunohistochemical analyses were cited by 3/12 post mortem analyses, while 2/12 relied on clinical history and pre-mortem symptoms. Serological testing was reported by 2/12 zoos, and 3/12 performed PCR on samples from live animals exhibiting clinical signs. Among the eight institutions that addressed the possibility of undiagnosed toxoplasmosis-related deaths, two acknowledged this as a potential concern, while one explicitly ruled it out. The remaining five considered the question not applicable to their context. Ten out of thirteen (10/13) institutions reported implementing additional preventive measures alongside vaccination. Only three identified vaccinations as their sole strategy. The most frequently cited complementary measures included the use of disinfectant footbaths (4/10), restricting the access of stray cats on zoo premises (3/10), washing vegetables (4/10), wearing gloves for food preparation (1/10), the use of protective masks (1/10), general hygiene practices (1/10), rodent control (3/10), and active disease surveillance in New World primates, macropods, and prosimians (1/10). All participants reported that their motivation to engage in the vaccination campaign was to prevent the clinical manifestations and/or mortality associated with toxoplasmosis. Twelve out of thirteen institutions stated that they would recommend the vaccine to other zoological facilities, while one abstained due to a lack of firsthand experience with the product at the time of the survey.

## 4. Discussion

Toxoplasmosis is frequently reported as one of the major causes of mortality in captive wildlife, and a rising trend in *T. gondii* cases in zoos was recently brought to the attention of the scientific community [9,13]. Currently, the only commercially available veterinary vaccine is Ovilis^®^ Toxovax (MSD Animal Health), a live attenuated vaccine which is not recommended for use in high-risk animals. As an alternative, VXN-Toxo, an inactivated vaccine administered intranasally has been developed and evaluated in various animal models, including mice [26], sheep [27], and non-human primates [28]. In a study assessing the protective effects of VXN-Toxo in *S. boliviensis* [28], a strong Th1-polarized immune response was observed, with no vaccine-associated antibody production. These findings underscore the critical role of cellular immunity, particularly IFN-gamma-mediated responses, in protection against toxoplasmosis [26].

The present work aimed to retrospectively compare the toxoplasmosis-associated mortality in captive animals before and after vaccination with VXN-Toxo. The vaccine was used to immunize various species across 29 zoos in Europe and the Americas, and among those zoos 20 participated in this study.

The vaccination not only protected seronegative animals from mortality, reducing it by 96.7%, but it might have also delayed or prevented the death of seropositive animals. Two out of seventeen *S. boliviensis* that received two vaccine doses at Bioparc of Doué-la-Fontaine were *T. gondii*-seropositive before the vaccination, and died of toxoplasmosis a year and two years after receiving the second dose. This is unusual, since sudden death is frequently observed in seropositive *Saimiri* in a short period after contracting the parasite [5]. It is possible that the vaccination might have prolonged the life of seropositive animals, and that subsequent booster immunizations might have delayed their death. The potential of the VXN-Toxo vaccine to reactivate memory T-cell response in a *T. gondii* seropositive *N. rufogriseus* has been previously described, suggesting a potential therapeutic application of the vaccine [31]. The most pronounced effect was observed after two doses of VXN-Toxo. The animal, chronically infected with *T. gondii* but asymptomatic at the time of vaccination, died two years later from unrelated causes. A post mortem examination revealed a single *T. gondii* cyst in the myocardium, with no evidence of systemic infection, indicating that parasite dissemination had been contained. It is worth mentioning that approximately one third of the wallabies vaccinated during the campaign were *T. gondii*-seropositive. The vaccine was also used in a therapeutic context in three seropositive *L. catta* at Parc d’Isle. These animals received the first dose during a toxoplasmosis outbreak, while already exhibiting clinical symptoms. Unfortunately, all three died before receiving the second dose. These cases suggest that while VXN-Toxo may be beneficial during chronic infection by preventing parasite reactivation, its efficacy during acute infection appears limited.

At least two doses might be necessary to obtain vaccine coverage. A case of death in an *L. catta* from the Exotic Garden shortly after the second dose of the vaccine suggests that the animal was likely infected before receiving the vaccine booster. This is consistent with previous findings in *S. boliviensis* [28] and seropositive wallabies [31], where two doses appear necessary to confer effective immunization, or reactivate protective immune responses in a seropositive animal. In contrast, a similar case at Parc d’Isle demonstrated a more favorable outcome. An *L. catta* contracted toxoplasmosis during a zoo outbreak after receiving the first vaccine dose but before the second. Clinical symptoms appeared approximately one week after the first booster. The animal was treated with clindamycin and metronidazole, gradually recovered, received the third vaccine dose, and has remained healthy to date, proving that the vaccine can potentially prevent mortality in seropositive lemurs.

Two death cases in *S. boliviensis* vaccinated at the Zoo and Botanical Garden of Mulhouse suggest that the immunity induced by VXN-Toxo may be time-limited and that sustained protection could require periodic booster immunizations. However, assessing the duration of vaccine-induced immunity is particularly challenging in the context of vaccination campaigns, which target rare and vulnerable species. Sampling for immune monitoring in these animals poses ethical and practical constraints, as it can induce significant stress and, in some cases, necessitates anesthesia. This procedure may increase the risk of opportunistic infections, as suspected in an *L. catta* case from Blérancourt. VXN-Toxo has been shown to induce a protective T-cell-mediated IFN-gamma response in vaccinated animals, without vaccine-associated antibody production [28]. Measuring cellular immunity is technically complex, and regular blood collection from zoo-housed animals is rarely feasible. Consequently, estimates of the duration of vaccine-induced protection remain indirect. The case of Pairi Daiza Zoo supports the effectiveness of the vaccine shortly after the completion of the three-dose regimen: all vaccinated animals remained protected during a subsequent outbreak, whereas unvaccinated individuals succumbed to the infection. This suggests that full vaccination confers strong, if potentially time-limited, protection against circulating *T. gondii* strains. Those examples of toxoplasmosis-related mortality emphasize the importance of reliable toxoplasmosis diagnosis in suspected cases, diligence during the vaccine administration (the proper use of the spray device for efficient vaccine administration, and assuring that the animal does not blow the vaccine out when sneezing), and regular booster immunization for animals at risk, especially when the presence of the parasite in suspected in the premises of the zoo.

A major limitation was the lack of comprehensive mortality data: not all zoos where vaccination was implemented perform standardized diagnostic procedures to determine causes of death. Among zoos that perform standardized diagnoses, the mortality in susceptible species was rarely constant, happening mostly during periodic outbreaks. Because of ethical reasons, only field trials are possible in those endangered species, and the efficacy of the vaccine against toxoplasmosis-related mortality could only be evaluated during outbreaks. Some of the zoos, like Amnéville Zoo and Bioparc of Doué-la-Fontaine, observed dramatic mortality in squirrel monkeys before the vaccination campaigns started (2005–2017), and the mortality was markedly reduced after the beginning of the vaccinations in December 2017. However, despite these institutions having a history of high and recurrent annual mortality due to toxoplasmosis, it is impossible to claim with certainty whether the lack of mortality was due to vaccine-induced protection or the absence of parasite exposure during the study period (2017–2025) in all of the studied zoos. Toxoplasmosis-related mortality in an unvaccinated animal was documented in 2024 in Bioparc of Doué-la-Fontaine, proving that the parasite was still present at their premises. A case in Pairi Daiza Zoo also provides compelling evidence of vaccine efficacy during an active outbreak. This Belgian Zoo experienced a toxoplasmosis outbreak, causing the death of sixteen *S. boliviensis*, which prompted them to search for additional preventive methods. Between 2021 and 2024, ninety-seven animals across seven species (Table 2)—primarily *S. boliviensis*, and *L. catta*—were vaccinated. In 2024, a new outbreak occurred. The only two unvaccinated *L. catta* died, while all vaccinated individuals, including those in the *Saimiri* genus, survived without incident. This outcome strongly supports the protective effect of VXN-Toxo under field conditions.

This outcome is particularly noteworthy given that some participating zoos in the program are located in South (Colombia, Brazil) or North America (Mexico), regions where *T. gondii* strain diversity differs markedly from Europe [32]. While type II and, to a lesser extent, I and III, are predominant in Europe and North America, South America is characterized by a broader diversity of circulating *T. gondii* strains, including a high proportion of atypical strains [33,34]. The reduction—and, in many cases, elimination—of toxoplasmosis-related deaths in both Europe and the Americas suggests that VXN-Toxo, based on an inactivated type I *T. gondii* strain (RH) formulated with NPLs, may confer broad protection, including against atypical strains commonly circulating in South America. Recently published data from the Barranquilla Zoo in Colombia further illustrate the vaccine’s impact [13]. Before the introduction of vaccination in 2022, the zoo experienced multiple toxoplasmosis-related deaths among neotropical primates and marsupials. Since 2022 in this Colombian zoo, the implementation of the vaccination program has been associated with the absence of reported cases of toxoplasmosis in vaccinated neotropical primates [13].

To date, toxoplasmosis management mainly consisted of meticulous hygiene practices, and as seen in the mortality cases presented in this paper, even the most diligent practices cannot guarantee the absence of outbreaks. Of all the zoos that participated in the survey on toxoplasmosis prevention, 75% employ practices other than vaccination to prevent the outbreaks. Of those practices, the most popular were footbaths, washing fruits and vegetables fed to the animals, limiting the presence of cats in the area, and rodent control. Of the 12 zoos that participated in the survey, 11 observed an improvement in toxoplasmosis prevention and declared their willingness to recommend the vaccine; one of them did not respond, as they have not yet used the vaccine.

## 5. Conclusions

This is the first time a vaccine has shown such consistent and broad-spectrum protection against *T. gondii* in zoological settings where different strains of the parasite were present and where the control of environmental exposure is inherently difficult. The reduction—and, in many cases, elimination—of toxoplasmosis-associated mortality following vaccination strongly supports VXN-Toxo as a highly promising prophylactic strategy. Its use could represent a turning point in toxoplasmosis prevention and management in zoos, offering a novel tool to protect endangered and sensitive species that are particularly susceptible to toxoplasmosis.

## Figures and Tables

**Table 1:** Animals vaccinated with VXN-Toxo in 20 European and American zoos between 2017 and 2025, with their respective International Union for Conservation of Nature (IUCN) conservation status.

Family	Common Name	Scientific Name	IUCN	Number	Adverse Effects Observed
Primates	Common squirrel monkey	*Saimiri sciureus*	LC	24	No
	Black-capped squirrel monkey	*Saimiri boliviensis*	LC	149	No
	Squirrel monkey	*Saimiri* spp.	LC	8	No
	Ring-tailed lemur	*Lemur catta*	EN	104	No
	Crowned lemur	*Eulemur coronatus*	EN	2	No
	Brown lemur	*Eulemur fulvus*	NT	12	No
	Black-and-white ruffed lemur	*Varecia variegata variegata*	CR	16	No
	Black lemur	*Eulemur macaco*	VU	2	No
	Red ruffed lemur	*Varecia variegata rubra*	CR	5	No
	Lac Alaotra Gentle Lemur	*Hapalemur alaotrensis*	CR	2	No
	White-faced saki	*Pithecia pithecia*	LC	8	No
	Cotton-top tamarin	*Saguinus oedipus*	CR	20	No
	Emperor tamarin	*Saguinus imperator*	LC	6	No
	Golden-handed tamarin	*Saguinus midas*	LC	8	No
	Geoffroy’s tamarin	*Saguinus geoffroyi*	NT	11	No
	White-footed tamarin	*Saguinus leucopus*	VU	10	No
	Golden-headed lion tamarin	*Leontopithecus chrysomelas*	EN	6	No
	Pied tamarin	*Saguinus bicolor*	CR	21	No
	Black tamarin	*Saguinus niger*	DD	1	No
	Brown-mantled tamarin	*Leontocebus fuscicollis*	LC	10	No
	Golden lion tamarin	*Leontopithecus rosalia*	EN	2	No
	Pygmy marmoset	*Cebuella pygmaea*	LC	31	No
	Geoffroy’s marmoset	*Callithrix geoffroyi*	LC	8	No
	Common marmoset	*Callithrix jacchus*	LC	6	No
	Goeldi’s marmoset	*Callimico goeldii*	VU	3	No
	White-cheeked spider monkey	*Ateles marginatus*	EN	4	No
	White-bellied spider monkey	*Ateles belzebuth*	EN	1	No
	Brown spider monkey	*Ateles hybridus*	CR	29	No
	Black-headed spider monkey	*Ateles fusciceps*	CR	23	No
	Black-faced black spider monkey	*Ateles chamek*	VU	2	No
	Southern muriqui/wooly spider monkey	*Brachyteles arachnoides*	EN	3	No
	Gray woolly monkey	*Lagothrix cana*	VU	3	No
	Colombian woolly monkey	*Lagothrix lagothricha*	VU	8	No
	White-handed gibbon	*Hylobates lar*	EN	2	No
	Howler monkey	*Alouatta caraya*	LC	5	No
	Venezuelan red howler monkey	*Alouatta seniculus*	LC	10	No
	White-fronted capuchin	*Cebus albifrons*	VU	11	No
	White-faced capuchin	*Cebus capucinus*	LC	8	No
	Tufted capuchin	*Sapajus apella*	LC	19	No
	Vieira’s titi monkey	*Plecturocebus vieirai*	VU	2	No
	Grey-necked night monkey	*Aotus lemurinus*	VU	3	No
	Northern night monkey	*Aotus trivirgatus*	DD	3	No
Marsupials	Red-necked/Bennett’s wallaby	*Notamacropus rufogriseus*	LC	33	No
	Swamp wallaby	*Wallabia bicolor*	LC	9	No
	Yellow-footed rock wallaby	*Petrogale xanthopus*	NT	18	No
	Parma wallaby	*Notamacropus parma*	VU	2	No
	Red kangaroo	*Osphranter rufus*	LC	14	No
	Western Grey kangaroo	*Macropus fuliginosus*	DD	4	No
	Bennett’s tree-kangaroo	*Dendrolagus bennettianus*	NT	16	No
	Eastern Bettong	*Bettongia gaimardi*	NT	6	No
	Brush-tailed rat-kangaroo	*Bettongia penicillata*	NT	3	No
	Long-nosed potoroo	*Potorous tridactylus*	NT	2	No
	Tasmanian wombat	*Vombatus ursinus*	LC	16	No
	Eastern quoll	*Dasyurus viverrinus*	EN	4	No
Felids	Pallas’ cat	*Otocolobus manul*	NT	2	No
Rodents	Azara’s agouti	*Dasyprocta azarae*	DD	3	No
Mustelidae	Small-clawed Asian otter	*Aonyx cinereus*	VU	5	No
Herpestidae	Meerkat	*Suricata suricatta*	LC	36	No
			Total number	784	

CR—Critically endangered, EN—Endangered, NT—Near Threatened, VU—Vulnerable, LC—Least Concern, DD—Data deficient. Potential vaccine-associated adverse effects were reported via detailed forms; the animals were screened for the presence of clinical signs like sneezing or runny nose, nose bleeding, watery eyes, anxiety and pain-related disorders, aggressiveness, lack or difficulty of movement, appetite loss, self-mutilation, unusual posture or activity, withdrawal from the group, unresponsiveness.

**Table 2:** Comparison of toxoplasmosis-related mortality in captive animals before and after vaccination with the VXN-Toxo vaccine.

Institution/ Zoo (Country)	Toxoplasmosis-Related Mortality Before the Vaccination		Vaccinations	Toxoplasmosis-Related Mortality After the Vaccination	
	Period	Dead Animals	Vaccinated Animals	Period	Details
Bioparc of Doué-la- Fontaine Zoo (France, Doué-la- Fontaine)	2005–2017	*S. boliviensis n* = 23 *L. catta n* = 1 (total *n* = 24)	*S. boliviensis n* = 17	2017–2025	*S. boliviensis n* = 4 (2 vaccinated twice, and 2 others were vaccinated while being seropositive; vaccine administration before the optimization of nasal spray delivery) *Procavia capensis n* = 1 (unvaccinated)
Amnéville Zoo (France, Amnéville)	2010–2017	*S. boliviensis n* = 33	*S. boliviensis n* = 9, *N. rufogriseus n* = 18 (total *n* = 27)	2017–2025	*N. rufogriseus n* = 1 (vaccinated while being seropositive, 3 doses)
Besançon Zoo (France, Besançon)	2010	*Petrogale* spp. *n* = 1	*S. boliviensis n* = 11	2018–2025	0
Parc d’Isle (France, Saint-Quentin)	2023	*N. rufogriseus n* = 10	*Saimiri* spp. *n* = 6, *L. catta n* = 13, *N. rufogriseus n* = 9, *S. suricatta n* = 4, *(total n = 32)*	2023–2025	*L. catta n* = 2, (vaccinated while showing signs of clinical toxoplasmosis)
The Exotic Garden, Foster’s Center For Small Primates (France, Blérancourt)	2023–2024	*L. catta n* = 4	*Saimiri* spp. *n* = 2 *L. catta n* = 5, *E. fulvus n* = 12, *C. jacchus n* = 6, *S. imperator n* = 4, *C. pygmaea n* = 4, *C. goeldii n* = 3, *S. midas n* = 3, *E. macaco n* = 2, *S. Oedipus n* = 2, *V. variegata variegata n* = 1 (total *n* = 44)	2024–2025	*L. catta*, *n* = 1 (2 doses)
La Palmyre Zoo (France, Les Mathes)	2013–2016	*S. suricata n* = 2	*S. sciureus n* = 6	2022–2025	0
Biotropica Zoo (France, La Coudrette)		unavailable	*S. boliviensis n* = 21	2023–2025	*N. parma n* = 2 (unvaccinated), and *n* = 1 suspected (unvaccinated) *M. fuliginosus n* = 1 (unvaccinated)
Zoo and Botanical Garden of Mulhouse (France, Mulhouse)	2017	*S. boliviensis n* = 4	*S. boliviensis n* = 5, *P. xanthopus n* = 14, *O. manul n* = 2 (total *n* = 21)	2017–2025	*S. boliviensis n* = 2, (5 doses of the vaccine and died 3–4 years after the last booster)
Pairi Daiza (Belgium, Brugelette)	2016	*S. boliviensis peruviensis n* = 16	*S. boliviensis peruviensis n* = 32, *L. catta n* = 25, *S. oedipus n* = 7, *P. pithecia n* = 6, *V. variegata variegata n* = 5, *E. coronatus n* = 2 (total *n* = 77)	2021–2025	*L. catta*, *n* = 2, unvaccinated
Apenheul Zoo (The Netherlands, Apeldoorn)	2021–2022	*A. seniculus n* = 2	*A. seniculus n* = 4, *P. pithecia n* = 2, *L. chrysomelas n* = 2, *S. bicolor n* = 1 (total *n* = 9)	2023–2025	0
Wildlands (The Netherlands, Emmen)	2012–2019	*S. boliviensis n* = 1, *L. catta n* = 12 (total *n* = 13)	*S. boliviensis n* = 21 *L. catta n* = 15, *W. bicolor n* = 5 *C. pygmaea n* = 4 *V. variegata n* = 2 (total *n* = 47)	2022–2025	0
Copenhagen Zoo (Denmark, Frederiksberg)	2014–2020	*L. catta n* = 4, *S. suricatta n* = 1, *M. giganteus n* = 2, *V. ursinus n* = 1 *Vulpes lagopus n* = 1 *Ovibos moschatus n* = 1 (total *n* = 10)	*L. catta n* = 11, *V. ursinus n* = 13, *P. xanthopus n* = 4, *D. viverrinus n* = 4, *B. penicillata n* = 3, *P. tridactylus n* = 2 (total *n* = 37)	2021–2025	*S. suricatta n* = 1, unvaccinated
Curraghs Wildlife Park (Isle de Man, Ballaugh)	2017–2020	*S. boliviensis peruviensis n* = 1 *S. suricatta n* = 1 *A. cinereus n* = 8 (total *n* = 10)	*S. boliviensis boliviensis n* = 7, *L. catta n* = 9, *S. suricatta n* = 11, *A. cinereus n* = 5 *D. azarae n* = 3, *A. fusciceps robustus n* = 2, *H. alaotrensis n* = 2, *V. variegata rubra n* = 2, *V. variegata variegata n* = 2 (total *n* = 43)	2023–2025	0
Budapest Zoo and Botanical Garden (Hungary, Budapest)	2013–2018	*Notamacropus eugenii n* = 5 *D. bennettianus n* = 3 *Capra aegagrus hircus n* = 1 (total *n* = 9)	*D. bennettianus n* = 16, *B. gaimardi n* = 6, *M. fuliginosus n* = 4 *V. ursinus n* = 3 *N. parma n* = 2 (total *n* = 31)	2023–2025	0
Lagos Zoo (Portugal, Lagos)	2018–2020	*S. boliviensis n* = 6 *L. catta n* = 3 (total *n* = 9)	*S. boliviensis n* = 26, *L. catta n* = 16, *S. suricatta n* = 5, *C. geoffroyi n* = 8, *V. variegata rubra n* = 3, *C. pygmaea n* = 6, *S. imperator n* = 2, *S. midas n* = 2, *S. oedipus n* = 2 *V. variegata variegata n* = 2, *W. bicolor n* = 4 *(total n = 76)*	2021–2025	*S. boliviensis n* = 2 (1 vaccinated, 1 of unknown status)
Africam Safari (Mexico, Puebla)	2012–2024	*S. sciureus n* = 4 *L. catta n* = 5 *N. rufogriseus n* = 20 *S. suricatta n* = 2 *O. rufus n* = 7 (total *n* = 38)	*O. rufus n* = 14 *S. suricatta n* = 5 (total *n* = 19)	2024–2025	0
Quinzinho de Barros Zoo (Brazil, Sorocaba)	2021–2024	*L. catta n* = 1 *B. arachnoides n* = 3 (total *n* = 4)	*L. catta n* = 3, *S. bicolor n* = 20, *A. caraya n* = 5, *L. chrysomelas n* = 4, *A. marginatus n* = 4, *S. midas n* = 3, *L. fuscicollis n* = 3, *L. cana n* = 3, *B. arachnoides n* = 3, *P. vieirai n* = 2, *A. chamek n* = 2, *A. trivirgatus n* = 2, *L. rosalia n* = 2, *A. trivirgatus n* = 1, *S. niger n* = 1 (total *n* = 58)	2024–2025	0
Conservacion Park of Medellín (Colombia, Medellín)	2015–2022	*-*	*S. sciureus n* = 9, *A. hybridus n* = 21, *C. albifrons n* = 11, *A. fusciceps n* = 8, *A. seniculus n* = 6, *S. geoffroyi, n* = 4 *C. capucinus n* = 4, *S. apella n* = 3, *S. oedipus n* = 5, *C. pygmaea n* = 1, *L. lagothricha n* = 1 (total *n* = 73)	2022–2025	0
Cali Zoo (Colombia, Cali)	2020–2023	*S. sciureus n* = 1 *L. catta n* = 1 *N. rufogriseus n* = 2 *C. pygmaea n* = 1 *S. oedipus n* = 1 *A. lemurinus n* = 2 *O. leucopus n* = 2 *L. lagothricha n* = 1 *V. variegata variegata n* = 1 (total *n* = 12)	*S. sciureus n* = 9, *L. catta n* = 7, *N. rufogriseus n* = 6, *S. suricatta n* = 11, *C. pygmaea n* = 7, *S. leucopus n* = 8, *L. lagothricha n* = 7, *S. apella n* = 7, *L. fuscicollis n* = 5, *C. capucinus n* = 4, *V. variegata variegata n* = 4, *A. fusciceps n* = 2, *H. lar n* = 2 (total *n* = 79)	2023–2025	*N. rufogriseus n* = 3. (2 unvaccinated and 1 vaccinated animal in which vaccine administration failure was suspected)
Baranquilla Zoo (Colombia, Barranquilla)	2007–2022	*N. rufogriseus n* = 2, *L. catta n* = 2, *V. variegata variegata n* = 1, *S. geoffroyi n* = 3, *S. oedipus n* = 1, *A. lemurinus n* = 1, *S. suricatta n* = 2 (total *n* = 12) [13]	*A. fusciceps n* = 11, *S. oedipus n* = 4, *L. fuscicollis n* = 2, *S. geoffroyi n* = 7, *S. apella n* = 9, *C. pygmaea n* = 9, *A. hybridus n* = 8, *A. lemurinus n* = 3, *A. belzebuth n* = 1, *S. leucopus n* = 2 (total *n* = 56)	2022–2025	
Total		214	784		23 ^A^, 7 ^B^

^A^ all the animals, including *T. gondii*-infected animals that were vaccinated post infection, ^B^ total number excluding animals that were seropositive to *T. gondii* prior to vaccination.

## Abbreviations

The following abbreviations are used in this manuscript:

EU	European Union
FVE	Federation of Veterinarians of Europe
NPLs	Lipid core maltodextrin nanoparticles
GMP	Good Manufacturing Processes
IHC	Immunohistochemistry
IUCN	International Union for Conservation of Nature
EAZA	European Association of Zoos and Aquaria
PCR	Polymerase Chain Reaction
